# Impact of different renal function equations on direct oral anticoagulant concentrations

**DOI:** 10.1038/s41598-021-03318-4

**Published:** 2021-12-13

**Authors:** Shin-Yi Lin, Ching-Hua Kuo, Tao-Min Huang, Yu-Fong Peng, Chih-Fen Huang, Sung-Chun Tang, Jiann-Shing Jeng

**Affiliations:** 1 Department of Pharmacy, National Taiwan University Hospital, Taipei, Taiwan; 2 School of Pharmacy, College of Medicine, National Taiwan University, Taipei, Taiwan; 3 Division of Nephrology, Department of Internal Medicine, National Taiwan University Hospital, Taipei, Taiwan; 4 Department of Neurology, Stroke Center, National Taiwan University Hospital, No. 7, ZhongShan South Road, Taipei, 100 Taiwan

**Keywords:** Cardiology, Medical research

## Abstract

The purpose of this study is to investigate the correlation between glomerular filtration rate (GFR) estimated by different renal function equations and non-vitamin K antagonist oral anticoagulant concentration. Atrial fibrillation patients who aged ≥ 20 years and used dabigatran, rivaroxaban, or apixaban for thromboembolism prevention were enrolled to collect blood samples and measure drug concentrations using ultra-high-performance liquid chromatography with tandem mass spectrometry. The GFR was estimated using the Cockroft–Gault formula (abbreviated as creatinine clearance, CrCL), Chronic Kidney Disease Epidemiology Collaboration formula (CKD-EPI) featuring both creatinine and cystatin C, and the Modification of Diet in Renal Disease Study equation (MDRD). Multivariate regression was used to investigate the associations of different renal function estimates with drug concentrations. A total of 511 participants were enrolled, including 146 dabigatran users, 164 rivaroxaban users and 201 apixaban users. Compared to clinical trials, 35.4% of dabigatran, 4.9% of rivaroxaban, and 5.5% of apixaban concentrations were higher than the expected range (*p* < 0.001). CKD-EPI and MDRD estimates classified fewer patients as having GFR < 50 mL/min than CrCL in all 3 groups. Both CrCL and CKD-EPI were associated with higher-than-expected ranges of dabigatran or rivaroxaban concentrations. Nevertheless, none of the renal function equations was associated with higher-than-expected apixaban concentrations. For participants aged ≥ 75 years, CKD-EPI may be associated with higher-than-expected trough concentration of dabigatran. In conclusion, CrCL and CKD-EPI both can be used to identify patients with high trough concentrations of dabigatran or rivaroxaban. Among elderly patients who used dabigatran, CKD-EPI may be associated with increased drug concentration.

## Introduction

Direct oral anticoagulants (DOAC) directly inhibit thrombin or factor Xa to achieve an anticoagulant effect^1^. Compared with warfarin, DOAC are non-inferior in preventing thromboembolism associated with atrial fibrillation (AF). In addition, DOAC have a comparable risk of major bleeding and a lower risk of intracranial hemorrhage than warfarin^2–4^. The extent of DOAC eliminated through the kidney varies. Dabigatran excretion is highly kidney-dependent, and approximately 80% of it is excreted unchanged via urine^5^. Both the Randomized Evaluation of Long-Term Anticoagulation Therapy (RE-LY) trial and real-world investigations have shown that the dabigatran concentration increased in patients with impaired renal function^6,7^. For rivaroxaban, approximately 66% of the drug was excreted in the urine, including 36% as an unchanged drug and 30% as an eliminated hepatic metabolite. For apixaban, the proportion of renal excretion of unchanged drug was only 27%^8^. Nevertheless, increased rivaroxaban and apixaban exposure was still noted in patients with profound renal impairment^9,10^.

The Cockcroft–Gault (C–G) formula is the most commonly used equation to estimate creatinine clearance (CrCL) and guide renal dose administration^11,12^. Dabigatran should be avoided in patients with CrCL less than 30 mL/min or undergoing dialysis according to the label in Taiwan^2,13^. However, there were no renal dose adjustment criteria in the RE-LY study. According to expert opinions, a reduced dosing regimen (i.e., 110 mg twice daily) is recommended among patients with CrCL rates between 30 and 50 mL/min^1,13,14^. For rivaroxaban, the dosage criteria in both the ROCKET AF (Rivaroxaban Once Daily Oral Direct Factor Xa Inhibition Compared with Vitamin K Antagonism for Prevention of Stroke and Embolism Trial in Atrial Fibrillation) trial and the J-ROCKET AF (Japanese Rivaroxaban Once Daily Oral Direct Factor Xa Inhibition Compared with Vitamin K Antagonism for Prevention of Stroke and Embolism Trial in Atrial Fibrillation) trial have been approved by the Taiwanese Food and Drug Administration. In patients with CrCL less than 50 mL/min, the rivaroxaban dose should be adjusted to 15 mg daily as per the ROCKET-AF trial and 10 mg daily as per the J-ROCKET AF trial^3,15^. For apixaban, the dose adjustment criteria are complicated and disregard CrCL. The dose should be cut in half (i.e., 2.5 mg twice daily) for patients who fulfill two of the following characteristics: age ≥ 80 years, weight < 60 kg, and serum creatinine level > 1.5 mg/dL^4^.

The creatinine assay used to develop the C–G method was not standardized and was likely 10–20% higher, leading to an incorrect estimate of renal function^11,16^. Since 2005, several equations to estimate GFR have been developed with standardized serum creatinine (CRE) assays, such as the Chronic Kidney Disease Epidemiology Collaboration (CKD-EPI) equation^17–19^ and the Modification of Diet in Renal Disease (MDRD) Study eqiation^20^. Using measured GFR as the gold standard, both the CKD-EPI and MDRD Study equations provided accurate estimations of GFR, in contrast to the C–G formula, while CKD-EPI had the best estimation^21^. The GFR estimated by the CKD-EPI equation is recommended by the Kidney Disease Improving Global Outcomes (KDIGO) guidelines to report kidney function^22^. In addition, compared to the MDRD study equation, the CKD-EPI equation has a higher correlation with cardiovascular risk and mortality^23^.

Several investigations have discussed the association between GFR estimated by different renal function equations and clinical outcomes of DOAC therapy. One New Zealand study showed that the CKD-EPI equation overestimated the dabigatran dose compared to the C–G formula and caused an increased risk of hemorrhagic events^24^. Another Taiwanese study showed that both the CKD-EPI and MDRD study equations overestimated GFR, led to inappropriate DOAC doses, and further attenuated the benefit of DOAC compared with warfarin in reducing major bleeding^25^. Nevertheless, these studies based on insurance databases did not include cystatin C to improve the precision of GFR estimates and lacked DOAC concentration data. The main purpose of our present study is to examine the correlation between DOAC concentration and the GFR estimated by different renal function equations, including the C–G formula; the CKD-EPI equation, which featured both CRE and cystatin C; and MDRD study equations and to investigate which estimation approach provides more precise identification of patients with increased drug concentrations.

## Results

### Patient demographic characteristics

**Figure 1:**
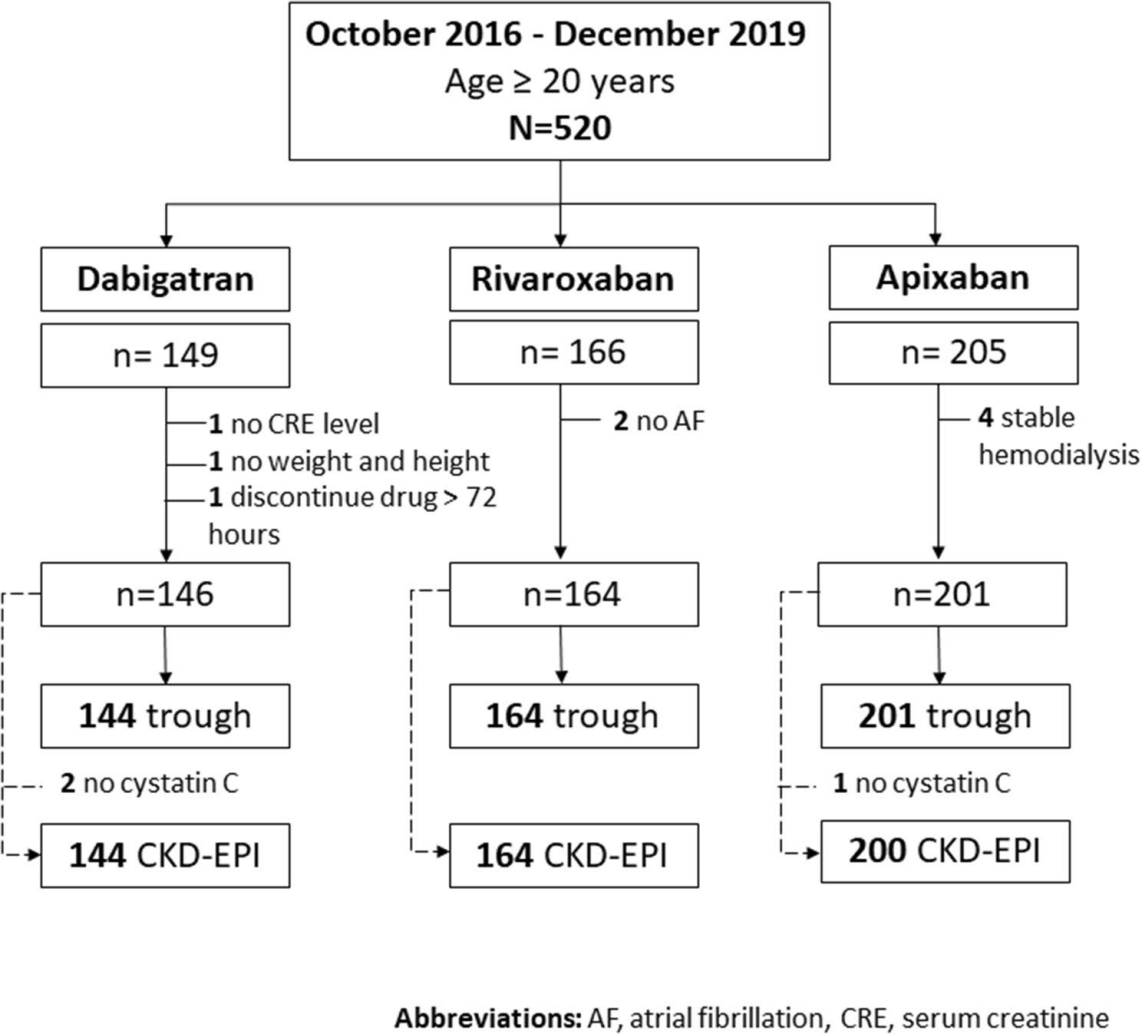
The study enrollment process.

A total of 520 participants were enrolled from October 2016 to December 2019. The participant enrollment process is depicted in Fig. 1. After applying the exclusion criteria, a total of 511 participants were included in the data analysis.

After excluding 2 participants with missing data for essential laboratory tests for GFR estimates, 1 participant who discontinued dabigatran for more than 3 days before drug level measurement, 2 participants in the rivaroxaban group who had no AF diagnosis, and 4 participants in the apixaban group who were on stable hemodialysis, 511 participants were included in the data analysis. Among them, 2 participants in the dabigatran group and 1 participant in apixaban group had missing data for cystatin C. Therefore, the CKD-EPI equation-estimated GFR was available only for the remaining 508 participants.

The average CrCL was 53.7 ± 19.7 mL/min. The average CKD-EPI was 65.4 ± 21.1, and the average MDRD was 64.9 ± 21.6 mL/min. Both CKD-EPI and MDRD were significantly higher than CrCL (*p* values both < 0.001). In addition, the number of participants with CKD-EPI < 50 mL/min was 126 (24.8%), the number with MDRD < 50 mL/min was 137 (26.8%), and the number with CrCL < 50 mL/min was 241 (47.2%). Both the CKD-EPI and MDRD Study equations classified fewer participants as having impaired renal function compared to the C–G formula (both *p* < 0.001).

A total of 146 (28.6%) participants took dabigatran, 164 (32.1%) took rivaroxaban, and 201 (39.3%) took apixaban. The comparisons for participants in the three groups are listed in Table 1. Compared to the remaining two groups, participants in the dabigatran group were more likely to be male, younger, and heavier; have better renal function reflected by lower CRE and cystatin C and higher GFR estimated by different renal function equations; have fewer congestive heart failure. Nevertheless, dabigatran users were more likely to have a previous stroke history. Regarding medication utilization, overall, 305 (59.7%) participants used a reduced dose regimen. Stratified by medication, more participants in the dabigatran group used a reduced dose (dabigatran 110 mg twice daily, 115 [78.8%]) than participants in the apixaban (2.5 mg twice daily, [57.2%]) and rivaroxaban (10 mg daily, [45.7%]) groups, and the difference was significant (*p* < 0.001).

**Table 1:** Comparison of basic characteristics of participants in the three medication groups.

	Overall N = 511	Dabigatran n = 146	Rivaroxaban n = 164	Apixaban n = 201	*p* value
**Demographic characteristics**					
Age (year)	75.3 ± 8.9	73.0 ± 9.0	75.0 ± 7.4	77.2 ± 9.7	< 0.001
≥ 75 years	259 (50.7)	59 (40.4)	74 (45.1)	126 (62.7)	< 0.001
Male	294 (57.5)	97 (66.4)	84 (51.2)	113 (56.2)	0.023
BW (kg)	64.8 ± 12.0	67.8 ± 11.4	63.4 ± 12.8	63.7 ± 11.4	0.001
BMI (kg/m^2^)	24.9 ± 3.9	25.3 ± 3.8	24.7 ± 4.2	24.8 ± 3.7	0.299
ALT (U/L)	21.2 ± 13.8	22.3 ± 15.8	20.0 ± 11.8	21.3 ± 13.7	0.619
CHA_2_DS_2_VASc^a^	4.1 ± 1.5	4.0 ± 1.4	3.9 ± 1.6	4.3 ± 1.6	0.071
HAS-BLED^b^	2.3 ± 0.9	2.4 ± 0.8	2.1 ± 0.8	2.4 ± 0.9	0.003
*Co-morbidities*					
IS or TIA	232 (45.4)	90 (61.6)	46 (28.0)	96 (47.8)	< 0.001
CHF	91 (17.8)	15 (10.3)	40 (24.4)	36 (17.9)	0.005
Hypertension	394 (77.1)	113 (77.4)	124 (75.6)	157 (78.1)	0.848
Diabetes	151 (29.5)	41 (28.1)	52 (31.7)	58 (28.9)	0.754
MI or PAOD	55 (10.8)	8 (5.5)	21 (12.8)	26 (12.9)	0.051
Malignancy	74 (14.5)	17 (11.6)	21 (12.8)	35 (17.4)	0.199
Bleeding history	76 (14.9)	20 (13.7)	19 (11.6)	37 (18.4)	0.170
ICH	16 (3.1)	5 (3.4)	2 (1.2)	9 (4.5)	0.200
GI bleeding	29 (5.7)	7 (4.8)	8 (4.9)	14 (7.0)	0.597
Other bleeding	33 (6.5)	8 (5.5)	11 (6.7)	14 (7.0)	0.846
**Renal function**					
CRE (mg/dL)	1.08 ± 0.37	0.98 ± 0.23	1.06 ± 0.32	1.17 ± 0.47	0.004
Cystatin C (mg/dL)	1.11 ± 0.36	1.04 ± 0.25	1.08 ± 0.35	1.18 ± 0.41	0.002
*eGFR (mL/min)*					
CrCL	53.7 ± 19.7	62.1 ± 19.6	51.9 ± 17.0	49.0 ± 20.1	< 0.001
≥ 30 to < 50 mL/min	199 (38.9)	45 (22.6)	67 (33.7)	87 (43.7)	< 0.001
< 30 mL/min	51 (10.0)	3 (5.9)	16 (31.4)	32 (62.7)	
*CKD-EPI*	65.4 ± 21.1	73.3 ± 19.5	64.7 ± 19.5	60.2 ± 21.8	< 0.001
≥ 30 to < 50 mL/min	104 (20.5)	21 (14.6)	30 (18.3)	53 (26.5)	0.001
< 30 mL/min	22 (4.3)	1 (0.7)	7 (4.3)	14 (7.0)	
*MDRD*	64.1 ± 21.6	72.4 ± 20.4	61.6 ± 19.0	60.0 ± 22.8	< 0.001
≥ 30 to < 50 mL/min	121 (23.7)	19 (13.0)	47 (28.7)	55 (27.4)	< 0.001
< 30 mL/min	16 (3.1)	0 (0)	3 (1.8)	13 (6.5)	
**DOAC utilization**					
*Trough concentration*	–	189.7 ± 168.8	45.2 ± 60.6	96.5 ± 55.2	–
Higher than expected range	70 (13.8)	51 (35.4)	8 (4.9)	11 (5.5)	< 0.001
Lower than expected range	76 (14.9)	13 (9.0)	45 (27.4)	18 (9.0)	< 0.001
*Medication use*^*c*^					
Standard dose	206 (40.3)	31 (21.2)	89 (54.3)	86 (42.8)	< 0.001
Reduced dose	305 (59.7)	115 (78.8)	75 (45.7)	115 (57.2)	
Suboptimal adherence^d^	51 (10.5)	14 (10.1)	9 (5.7)	28 (14.7)	0.023
*Concurrent medications*^*e*^					
Amiodarone	111 (21.7)	26 (17.8)	28 (17.1)	57 (28.4)	0.014
Dronedarone	17 (3.3)	1 (0.7)	7 (4.3)	9 (4.5)	0.108
Verapamil	9 (1.8)	2 (1.4)	0 (0)	7 (3.5)	0.039
NSAID	12 (2.3)	6 (4.1)	5 (3.0)	1 (0.5)	0.070
Aspirin	10 (2.0)	4 (2.7)	2 (1.2)	4 (2.0)	0.627
Clopidogrel	11 (2.2)	3 (2.1)	3 (1.8)	5 (2.5)	0.907

Data are expressed as mean ± standard deviation or number (percentage). A total of 146 participants were enrolled to dabigatran group and contributed 144 dabigatran trough concentrations. A total of 164 participants were enrolled to rivaroxaban group and 201 to apixaban group, and all contributed trough concentrations.

^a^CHA_2_DS_2_VASc score: To evaluate the risk for ischemic stroke among patients with atrial fibrillation. Higher score indicates higher risk of ischemic stroke. One point is assigned to congestive heart failure, hypertension, diabetes, age 65–74 years, female sex, or vascular disease and two points were assigned to age ≥ 75 years and history of ischemic stroke or transient ischemic attack.

^b^HASBLED score: To evaluate the risk for bleeding. Higher score indicates higher risk. One point is assigned to hypertension, abnormal liver function, abnormal renal function, stroke history, bleeding history, labile international normalized ratio (INR) during warfarin therapy, age over 65 years, antiplatelet agent, non-steroidal anti-inflammatory drug or ethanol use. The item labile INR was not calculated in the present study.

^c^Standard dose: 150 mg twice daily for dabigatran, 15 mg daily for rivaroxaban and 5 mg twice daily for apixaban; reduced dose: 110 mg twice daily for dabigatran,10 mg daily for rivaroxaban and 2.5 mg twice daily for apixaban.

^d^Suboptimal adherence was defined as no self-reported missed direct oral anticoagulant dose during 7 days before drug concentration monitoring. A total of 26 patients had missed data.

^e^Concurrent medications: None of the participants used azole antifungal agents, protease inhibitors, rifampin, and enzyme inducing antiepileptic drugs such as phenytoin, phenobarbital and carbamazepine.

*BMI* body mass index, *BW* body weight, *CKD-EPI* glomerular filtration rate estimated by using the chronic kidney disease epidemiology collaboration equation featured both creatinine and cystatin C, *CHF* congestive heart failure, *CrCL* creatinine clearance estimated by using the Cockroft–Gault formula, *CRE* serum creatinine, *DE* dabigatran etexilate, *eGFR* estimated glomerular filtration rate, *GI* gastrointestinal, *ICH* intracranial hemorrhage, *IS* ischemic stroke, *MDRD* the GFR estimated by using the modification of diet in renal disease (MDRD) study equation, *MI* myocardial infarction, *NSAID* non-steroidal anti-inflammatory drugs, *PAOD* peripheral arterial vascular disease, *TIA* transient ischemic attack.

**Figure 2:**
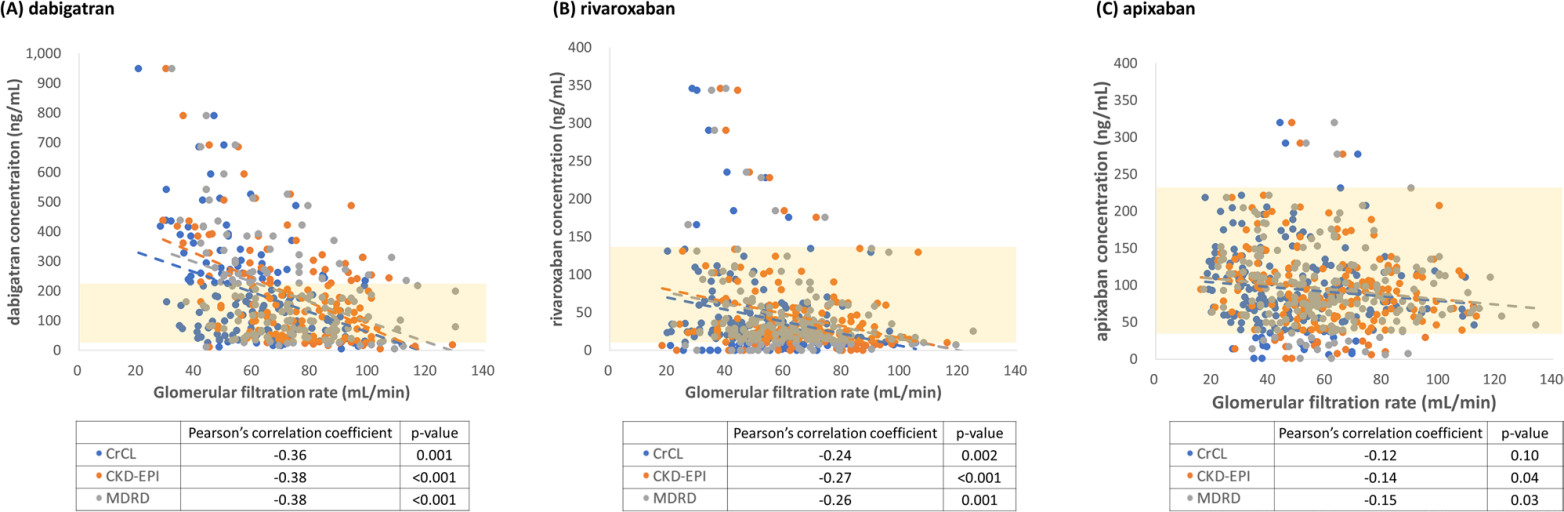
Distribution of drug concentration according to glomerular filtration rate estimated by different renal function equations: (**A**) dabigatran, (**B**) rivaroxaban and (**C**) apixaban.

The distribution of drug concentrations is depicted in Fig. 2. Participants in the dabigatran group were more likely to have higher-than-expected-range trough concentrations than those in the rivaroxaban or apixaban group (dabigatran, 51 [35.4%], rivaroxaban, 8 [4.9%], and apixaban 11 [5.5%] participants, *p* < 0.001). In contrast, participants who used rivaroxaban were more likely to have lower-than-expected-range trough concentrations than dabigatran and apixaban participants (dabigatran, 13 [9.0%]; rivaroxaban, 45 [27.4%]; and apixaban, 18 [9.0%]; *p* < 0.001).

The correlation between GFR and drug concentration is depicted in Fig. 2. The GFR was best associated with dabigatran concentration, followed by rivaroxaban and then apixaban, regardless of which formula was used. The Pearson’s correlation coefficients between each GFR estimate and the dabigatran, rivaroxaban, and apixaban concentration were listed in Fig. 2.

### Factors affecting high drug concentration

The characteristics of participants with higher-than-expected DOAC concentrations are listed in Table S2. Compared to the rest of the participants, those with increased DOAC concentrations had worse renal function, represented by higher CRE and lower CrCL in all 3 groups. In addition to renal function, dabigatran users with high concentrations were more likely to be female, older, and thinner, but were less likely to have ischemic stroke history, and had lower HAS-BLED score.

The results of multivariate logistic regressions are presented in Table 2. To predict higher-than-expected-range dabigatran concentrations, dabigatran dose and GFR reached the level of significance, regardless of which renal function equation was used. The associations between high dabigatran concentration and GFR were inverse (odds ratio [OR] and 95% confidence interval [CI] for CrCL, 0.97 [0.94, 1.00], *p* = 0.03, CKD-EPI 0.96 [0.94, 0.99], *p* = 0.002 and MDRD, 0.97 [0.95, 0.99], *p* = 0.01, respectively).

**Table 2:** Multivariate logistic regression for higher-than-expected-range drug concentrations.

Medication	OR and 95% CI	*p* value	OR and 95% CI	*p* value	OR and 95% CI	*p* value
**Dabigatran**						
Age (years)	1.04 (0.98, 1.10)	0.22	1.04 (0.98, 1.10)	0.24	1.07 (1.01, 1.12)	0.02
Male sex	0.43 (0.18, 1.02)	0.06	0.57 (0.23, 1.41)	0.22	0.50 (0.21, 1.23)	0.13
Weight (kg)	0.99 (0.95, 1.03)	0.61	0.99 (0.95, 1.03)	0.47	0.98 (0.95, 1.02)	0.40
Dabigatran dose (mg)	1.04 (1.01, 1.06)	0.01	1.04 (1.02, 1.07)	0.002	1.04 (1.01, 1.06)	0.01
eGFR	CrCL		CKD-EPI		MDRD	
	0.97 (0.94, 1.00)	0.03	0.96 (0.94, 0.99)	0.002	0.97 (0.95, 0.99)	0.01
**Rivaroxaban**						
Age (years)	0.97 (0.84, 1.12)	0.68	1.00 (0.87, 1.15)	1.00	1.04 (0.91, 1.19)	0.59
Male sex	0.30 (0.04, 2.23)	0.24	0.33 (0.04, 2.60)	0.30	0.36 (0.05, 2.73)	0.32
Weight (kg)	1.06 (1.00, 1.13)	0.07	1.03 (0.97, 1.11)	0.32	1.05 (0.98, 1.12)	0.14
rivaroxaban dose (mg)	1.35 (0.88, 2.06)	0.17	1.43 (0.91, 2.26)	0.12	1.33 (0.87, 2.04)	0.19
Amiodarone use	6.54 (0.94, 45.45)	0.06	6.11 (0.89, 41.69)	0.07	6.09 (0.89, 41.70)	0.07
Dronedarone use	41.36 (2.86, 598.77)	0.01	49.67 (2.75, 896.84)	0.01	37.40 (2.51, 558.36)	0.01
eGFR	CrCL		CKD-EPI		MDRD	
	0.89 (0.81, 0.97)	0.01	0.91 (0.85, 0.98)	0.01	0.91 (0.84, 0.98)	0.01
**Apixaban**						
Age (years)	1.03 (0.93, 1.14)	0.55	1.04 (0.95, 1.15)	0.38	1.05 (0.96, 1.16)	0.31
Male sex	13.54 (1.51, 121.32)	0.02	17.77 (1.87, 168.81)	0.01	15.63 (1.71, 143.27)	0.02
Weight (kg)	0.97 (0.90, 1.05)	0.46	0.96 (0.88, 1.04)	0.27	0.96 (0.89, 1.04)	0.34
apixaban dose (mg)	1.11 (0.53, 2.29)	0.79	1.22 (0.57, 2.61)	0.60	1.11 (0.53, 2.32)	0.78
eGFR	CrCL		CKD-EPI		MDRD	
	0.96 (0.91, 1.02)	0.21	0.97 (0.93, 1.01)	0.11	0.97 (0.94, 1.01)	0.19

*CI* confidence interval, *CKD-EPI* glomerular filtration rate estimated by using the chronic kidney disease epidemiology collaboration equation (CKD-EPI) featured both creatinine and cystatin C, *CrCL* creatinine clearance estimated by using the Cockroft–Gault formula, *eGFR* estimated glomerular filtration rate, *MDRD* the GFR estimated by using the modification of diet in renal disease (MDRD) study equation, *OR* odds ratio.

To predict higher-than-expected-range rivaroxaban concentrations, similar to dabigatran, GFR reached the level of significance, regardless of which renal function equation was applied (ORs for CrCL, CKD-EPI and MDRD, 0.89 [0.81, 0.97], 0.91 [0.85, 0.98] and 0.91 [0.84, 0.98], *p* all = 0.01, respectively). Concurrent use of dronedarone was also associated with high rivaroxaban concentration, but the 95% CI for the OR was wide due to the small number of participants (7 participants used dronedarone and 2 had higher-than-expected rivaroxaban concentrations).

To predict a higher-than-expected-range apixaban concentration, none of the GFR estimates reached the level of significance (ORs for CrCL, CKD-EPI and MDRD, 0.96 [0.91, 1.02], *p* = 0.21, 0.97 [0.93, 1.01], *p* = 0.11, and 0.97 [0.94, 1.01], *p* = 0.19, respectively). Furthermore, male sex was significantly associated with a high apixaban concentration.

For all the multivariate logistic regression models, the *p* value of the Hosmer and Lemeshow Test were > 0.05, which indicated a good model fit. In addition, none of the covariates in the multivariate logistic regression model displayed a VIF value > 10, which indicated no collinearity existed between variables.

### Subgroup analysis

We then classified participants into elderly (age ≥ 75 years) and non-elderly groups. We repeated the analysis to investigate whether the association between high drug concentrations and various GFR estimates was different in elderly individuals. The results are displayed in Table 3. For dabigatran, CKD-EPI were significantly associated with high dabigatran concentration (OR = 0.95 [0.91, 0.99], *p* = 0.01), but not CrCL or MDRD (ORs = 0.96 [0.92, 1.01], *p* = 0.13 for CrCL and 0.97 [0.94, 1.00], *p* = 0.08 for MDRD). However, the *p* value for interaction was not significant for all 3 GFR estimates (CrCL, *p* = 0.36, CKD-EPI, *p* = 0.16, MDRD, *p* = 0.48, respectively).

**Table 3:** Multivariate logistic regression for higher-than-expected-range drug concentrations in age subgroups.

Medication	Dabigatran (n = 59)		Rovaroxaban (n = 74)		Apixaban (n = 126)	
eGFR	OR (95% CI)	*p* value	OR (95% CI)	*p* value	OR (95% CI)	*p* value
**Age ≥ 75 years**						
CrCL	0.96 (0.92, 1.01)	0.13	0.87 (0.73, 1.02)	0.09	0.97 (0.89, 1.05)	0.45
CKD-EPI	0.95 (0.91, 0.99)	0.01	0.90 (0.79, 1.02)	0.11	0.98 (0.93, 1.03)	0.41
MDRD	0.97 (0.94, 1.00)	0.08	0.89 (0.78, 1.01)	0.07	0.98 (0.93, 1.03)	0.43

Medication	Dabigatran (n = 85)		Rovaroxaban (n = 90)		Apixaban (n = 75)	
eGFR	OR (95% CI)	*p* value	OR (95% CI)	*p* value	OR (95% CI)	*p* value
**Age < 75 years**						
CrCL	0.96 (0.92, 1.01)	0.09	0.88 (0.68, 1.14)	0.33	0.92 (0.81, 1.04)	0.19
CKD-EPI	0.97 (0.93, 1.01)	0.10	0.18 (0.00, 7.36)	0.36	0.94 (0.85, 1.03)	0.19
MDRD	0.97 (0.94, 1.00)	0.08	0.85 (0.62, 1.18)	0.34	0.95 (0.86, 1.04)	0.24

*CI* confidence interval, *CKD-EPI* glomerular filtration rate estimated by using the chronic kidney disease epidemiology collaboration equation (CKD-EPI) featured both creatinine and cystatin C, *CrCL* creatinine clearance estimated by using the Cockroft–Gault formula, *eGFR* estimated glomerular filtration rate, *MDRD* the GFR estimated by using the modification of diet in renal disease (MDRD) study equation, *OR* odds ratio.

For rivaroxaban, the association between different GFR estimates and high drug concentration were not significant, either in the elderly, or in the non-elderly groups. For apixaban, the results were similar with rivaroxaban, none of the GFR estimates were associated with high drug concentration in both age subgroups.

In addition, for dabigatran, dose was associated with high drug concentration. Therefore, we repeated the multivariate regression in participants using 110 mg regimen (115 participants). The results showed that reduced GFR was the only factor associated with high dabigatran concentration, regardless of which formula was used (ORs for CrCL, CKD-EPI and MDRD, 0.95 [0.92, 0.99], *p* = 0.02, 0.94 [0.91, 0.98], *p* = 0.002, and 0.96 [0.93, 0.99], *p* = 0.01, respectively). However, the interaction *p* value was not significant for all GFR estimates (CrCL, *p* = 0.57, CKD-EPI, *p* = 0.12, MDRD, *p* = 0.79, respectively). All logistic regression models for subgroup analyses displayed goodness-of-fit and none of the covariates in the model showed collinearity.

## Discussion

The present investigation provides real-world data on the associations between different GFR estimates and DOAC exposure in Asians, especially considering cystatin C-based renal function estimates. Both CrCL and CKD-EPI predicted increased concentrations of dabigatran and rivaroxaban, but not apixaban. Among elderly patients aged ≥ 75 years, CKD-EPI may predict high dabigatran concentrations.

Based on previous observations, elderly and female patients tended to have a higher estimated GFR based on the CKD-EPI and MDRD Study equation than the C–G formula^24–26^. In this investigation, the results were similar. The CKD-EPI and MDRD were significantly higher than CrCL; approximately 25% of the participants in the CrCL < 50 mL/min group were reclassified to an estimated GFR ≥ 50 mL/min based on the CKD-EPI or the MDRD equation. This was probably related to the high proportion of elderly participants: 50.2% of our participants were older than 75 years of age. According to pharmacokinetic studies, the proportion of renal elimination for dabigatran was highest, followed by rivaroxaban and apixaban^5,8,9^. Our real-world concentration data also showed that the correlation between drug concentration and GFR was best for dabigatran, followed by rivaroxaban and apixaban. Similarly, reduced GFR predicted high concentrations of dabigatran and rivaroxaban, but not apixaban. Although the CKD-EPI equation showed improved classification of renal function compared with the C–G formula, the two equations displayed comparable sensitivity in detecting increased dabigatran and rivaroxaban exposure.

Most physicians in Asia preferred a reduced dose regimen due to concerns regarding anticoagulant-related bleeding^27^. Although the majority of the participants in the dabigatran group received a reduced dose regimen (80%), approximately 30% of these patients still had a higher-than-expected-range dabigatran concentration. Furthermore, our data showed that dabigatran dose also predicted concentrations higher than the expected range. To eliminate the influence of dose, we specifically analyzed participants using the 110-mg regimen and found that GFR was the only factor to predict increased dabigatran concentration. This finding reaffirmed the important role of renal function in dabigatran exposure.

We also focused specifically on elderly participants and found that the CKD-EPI equation, but not the C–G formula, detected patients with a risk of increased dabigatran concentration. Elderly patients are more likely to suffer from sarcopenia and reduced muscle mass, which causes lower CRE production and imprecise GFR estimation by the C–G formula^28^. The CKD-EPI equation used in our investigation considered both CRE and cystatin C^18^. Cystatin C is unaffected by muscle mass and age^28^. Adding cystatin C on top of CRE increases the precision of the GFR estimation^21^. A previous investigation also showed that CKD-EPI explained a higher proportion of the variation in dabigatran concentration than the C–G formula^29^. In addition, in the RE-LY trial, the GFR estimated by the CKD-EPI equation ≥ 80 mL/min predicted lower major and life-threatening bleeding risk but not CrCL^7,12^. Nevertheless, the *p* value for the interaction term did not reach the level of significance, which may be attributed to small sample size. The result should be interpreted with caution.

Similar to previous data, the proportion of rivaroxaban and apixaban concentrations with a higher-than-expected-range were low, ranged from 5–6%^30^. Furthermore, all of the rivaroxaban users in our study followed J-ROCKET AF dosage criteria, and an off-label underdosing regimen was not uncommon. A total of 13.4% of rivaroxaban users and 29.9% of apixaban users were prescribed lower dosages than the recommended dosing regimen. These findings may explain why the standard dose regimen did not predict increased DOAC concentrations. Nevertheless, GFR was still an essential predictor for high rivaroxaban concentrations. In addition, concurrent use of dronedarone predicted high rivaroxaban concentration., which was well correlated with the mechanism of drug interaction. Dronedarone inhibits *p*-glycoprotein and causes increased rivaroxaban exposure^31^. For apixaban, due to the low proportion of renal excretion, the impact of GFR on drug concentration was nonsignificant. Interestingly, male sex increased the risk of increased apixaban exposure. The same finding has been mentioned in a previous investigation^30^. The reason behind this observation was not clear. One explanation may be that male patients are less likely to fulfill the dose adjustment criteria of apixaban.

From our data, the proportion of participants with suboptimal adherence was approximately 10%. Although the proportion of participants with suboptimal adherence was significantly lower in the rivaroxaban group than in the dabigatran or apixaban group, the nonrandomized design of our study was unable to conclude whether this observation resulted from the frequency of administration. Nevertheless, the correlation between suboptimal DOAC adherence and low drug concentration has been stressed before^6^. In the multivariate regression in the present study, suboptimal adherence also predicted low dabigatran and apixaban concentrations. Therefore, comprehensive patient education to improve DOAC adherence is important. Overall, measurement of DOAC concentrations in specific populations may be beneficial because it provides physicians with information about the status of DOAC exposure, helps them to make clinical judgments and improves the efficacy and safety of DOAC therapy.

The present study investigated real-world data on dabigatran, rivaroxaban and apixaban concentrations among Asians and reported the associations of different GFR estimates with drug exposure. We also incorporated cystatin C for GFR estimation, which improved the precision. Nevertheless, our study has the following limitations. First, this is an observational study and we did not collect iohexol clearance as the standard reference of GFR. Therefore, we were not able to provide the accuracy of our GFR estimations. In addition, the GFR values estimated by different renal function equations were highly correlated. It is difficult to identify differences among equations in predicting high or low drug concentrations based on our study size. However, cystatin C-based renal function estimates may be associated with high drug concentrations in elderly individuals than CrCL. Future larger-scale studies which included measurement of GFR reference standard are necessary to validate our data. Second, the patient characteristics in our investigation is different from that in clinical trials. This can be the cause for discrepancy of the DOAC concentration with the expected range reported in trials. However, currently, the therapeutic range for DOAC is not clear. Using the expected range as a surrogate of reference for comparison helps us identifying factors causing DOAC concentration difference in real world practice. Third, the dabigatran concentration varied widely, and some trough concentrations even reached the range for peak concentration. These outlier data can influence the correlation between dabigatran concentration and eGFR. Potential causes of these data included low eGFR, using 150 mg regimen of dabigatran, and drug interaction. The wide inter-individual variation of dabigatran concentration echoed the importance of DOAC concentration measurement among specific population. Last, we did not include clinical outcomes, instead using drug concentration as a surrogate for drug exposure. Previous data showed that the overestimation of renal function by the CKD-EPI equation led to an increased risk of bleeding because higher dosing regimens were prescribed^24,25^. Further larger-scale studies with longer follow-up durations are warranted to address the associations between drug concentration and clinical outcomes.

In conclusion, our data showed that both the C–G formula and the CKD-EPI equation both predicted higher-than-expected-range dabigatran and rivaroxaban concentrations. In elderly individuals aged over 75 years receiving dabigatran therapy, the CKD-EPI equation, but not the C–G formula, predicted increased drug levels.

## Methods

### Study design

This prospectively enrolled study was conducted at National Taiwan University Hospital (NTUH) in Taiwan. Patients aged over 20 years, diagnosed with AF, and prescribed dabigatran, rivaroxaban or apixaban for more than 7 days fulfilled the criteria of inclusion, regardless of whether they were outpatients or inpatients. Patients who were under stable hemodialysis or peritoneal dialysis, were pregnant or breastfeeding, had contraindications for DOAC therapy, or did not provide written informed consent were excluded. The study protocol was approved by the Institutional Ethics Committee of NTUH, and all study processes were performed in accordance with relevant guidelines and regulations. Each participant was required to provide written informed consent for participation in the study.

### Plasma DOAC concentration analysis

Blood samples were collected through venous puncture and stored in tubes containing K2EDTA (BD Vacutainer®). The date of DOAC concentration measurement was defined as the index date. The trough concentration was measured immediately before the next dose of dabigatran, rivaroxaban or apixaban. Plasma dabigatran, rivaroxaban, or apixaban concentrations were measured using ultra-high-performance liquid chromatography with tandem mass spectrometry (UHPLC-MS/MS). The DOAC concentrations were compared with those in the data reported in clinical trials. The expected trough dabigatran concentration ranged from 28 to 215 ng/mL, the expected trough rivaroxaban concentration ranged from 12 to 137 ng/mL, and the expected range for trough apixaban concentration ranged from 34 to 230^1,5,8,9,14^. All plasma drug concentrations were classified to be higher, within, or lower than the expected range, according to the aforementioned value. A detailed description of the method of plasma DOAC concentration analysis is described in the supplementary material.

### Clinical data acquisition

Baseline participant characteristics were manually retrieved from electronic medical records. The following details were recorded: (1) participant characteristics; (2) laboratory tests, the one which was nearest to the index date; (3) comorbid diseases from the records of outpatient, inpatient and emergency department within 5 years from the index date; (4) DOAC prescription details on the index date; (5) concurrent medications showing potential interactions with DOAC on the index date: antiplatelet agents, including aspirin, clopidogrel, prasugrel, ticagrelor, and cilostazol; nonsteroidal anti-inflammatory agents (as-needed use was excluded); amiodarone; dronedarone; azole antifungal agents; macrolide antibiotics; enzyme-inducing antiepileptic drugs (AEDs) including phenytoin, phenobarbital, and carbamazepine; and other AEDs such as levetiracetam and valproic acid. The risk of thromboembolism was evaluated using the CHA_2_DS_2_-VASc score^32^ and the risk of bleeding was evaluated using the HAS-BLED score^33^.

### Estimation of GFR

Different formulae were used to estimate the GFR. The details of formulae and abbreviations used in this study are presented in Table S1, including the C–G formula^11^, CKD-EPI formulae featuring both CRE and cystatin C^18^, and the MDRD Study equation^20^. The GFRs estimated by these equations were abbreviated as CrCL, CKD-EPI and MDRD. The estimates provided by the MDRD study equation and CKD-EPI equation were normalized to a body surface area (BSA) of 1.73 mL/min/m^2^. To consider the effect of body size on GFR estimates, we adjusted the results based on each patient’s BSA.

### Measurement of DOAC adherence

All participants were administered a questionnaire to measure DOAC adherence. Suboptimal adherence was defined as any missed dose over the past week.

### Statistical analysis

The main analysis of this investigation is cross-sectional, despite the prospective enrollment design. The creatinine or cystatin C value nearest to the index date was used to estimate GFR to investigate the association with out-of-expected DOAC concentrations. Mean, standard deviation, median, and interquartile range (IQR) were used to summarize the descriptive analyses. Intergroup differences were compared using Student’s t tests, Mann–Whitney U tests, chi-squared tests, or analysis of variance, as appropriate. To investigate factors associated with higher-than-expected DOAC concentration, univariate logistic regression was used first to identify factors associated with high DOAC concentration, defined as a *p* value of < 0.05. Further, clinically significant factors included age, sex, weight, DOAC dose regimen and eGFR estimated with different renal function equations were also adjusted in the model of multivariate logistic regression. Hosmer and Lemeshow Test was used to test goodness-of-fit for the multivariate logistic regression models. In addition, the collinearity of variables in the model was test by using liner regression. Variables with VIF > 10 was considered to have collinearity with other variables. Pearson’s or Spearman’s correlation was further applied to identify the variables with collinearity. Data were analyzed using IBM SPSS Statistics (version 26.0; IBM Corp., Armonk, NY, USA). The level of statistical significance was set at 0.05.

## Supplementary Information

Supplementary Information 1.

Supplementary Table S1.

Supplementary Table S2.

## Data Availability

Data are available upon request.

## References

[1] Steffel J (2021). European heart rhythm association practical guide on the use of non-vitamin K antagonist oral anticoagulants in patients with atrial fibrillation. Europace Eur. Pacing Arrhythm. Card. Electrophysiol. J. Work. Groups Card. Pacing rrhythm. Card. Electrophysiol. Eur. Soc. Cardiol..

[2] Connolly SJ (2009). Dabigatran versus warfarin in patients with atrial fibrillation. N. Engl. J. Med..

[3] Patel MR (2011). Rivaroxaban versus warfarin in nonvalvular atrial fibrillation. N. Engl. J. Med..

[4] Granger CB (2011). Apixaban versus warfarin in patients with atrial fibrillation. N. Engl. J. Med..

[5] Stangier J (2008). Clinical pharmacokinetics and pharmacodynamics of the oral direct thrombin inhibitor dabigatran etexilate. Clin. Pharmacokinet..

[6] Lin SY (2019). Factors affecting serum concentration of dabigatran in Asian patients with non-valvular atrial fibrillation. J. Formos. Med. Assoc. Taiwan yi zhi..

[7] Reilly PA (2014). The effect of dabigatran plasma concentrations and patient characteristics on the frequency of ischemic stroke and major bleeding in atrial fibrillation patients: The RE-LY trial (randomized evaluation of long-term anticoagulation therapy). J. Am. Coll. Cardiol..

[8] Byon W, Garonzik S, Boyd RA, Frost CE (2019). Apixaban: A clinical pharmacokinetic and pharmacodynamic review. Clin. Pharmacokinet..

[9] Mueck W, Stampfuss J, Kubitza D, Becka M (2014). Clinical pharmacokinetic and pharmacodynamic profile of rivaroxaban. Clin. Pharmacokinet..

[10] Stanifer JW (2020). Apixaban versus warfarin in patients with atrial fibrillation and advanced chronic kidney disease. Circulation.

[11] Cockcroft DW, Gault MH (1976). Prediction of creatinine clearance from serum creatinine. Nephron.

[12] Hijazi Z (2014). Efficacy and safety of dabigatran compared with warfarin in relation to baseline renal function in patients with atrial fibrillation: A RE-LY (randomized evaluation of long-term anticoagulation therapy) trial analysis. Circulation.

[13] Lin SY, Tang SC, Shen LJ, Jeng JS (2015). Prothrombin complex concentrate (Beriplex P/N)-related renal and cerebral infarctions in a patient with warfarin-associated intracerebral hemorrhage. J. Stroke Cerebrovasc. Dis..

[14] Steffel J (2018). The 2018 european heart rhythm association practical guide on the use of non-vitamin K antagonist oral anticoagulants in patients with atrial fibrillation. Eur. Heart J..

[15] Hori M (2012). Rivaroxaban vs. warfarin in Japanese patients with atrial fibrillation—the J-ROCKET AF study. Circ. J. Off. J. Jpn. Circ. Soc..

[16] Myers GL (2006). Recommendations for improving serum creatinine measurement: A report from the laboratory working group of the national kidney disease education program. Clin. Chem..

[17] Levey AS (2009). A new equation to estimate glomerular filtration rate. Ann. Intern. Med..

[18] Inker LA (2012). Estimating glomerular filtration rate from serum creatinine and cystatin C. N. Engl. J. Med..

[19] Inker LA (2011). Expressing the CKD-EPI (chronic kidney disease epidemiology collaboration) cystatin C equations for estimating GFR with standardized serum cystatin C values. Am. J. Kidney Off. J. Natl. Kidney Found..

[20] Levey AS (2006). Using standardized serum creatinine values in the modification of diet in renal disease study equation for estimating glomerular filtration rate. Ann. Intern. Med..

[21] Michels WM (2010). Performance of the Cockcroft–Gault, MDRD, and new CKD-EPI formulas in relation to GFR, age, and body size. Clin. J. Am. Soc. Nephrol. CJASN.

[22] Stevens PE, Levin A (2013). Evaluation and management of chronic kidney disease: Synopsis of the kidney disease: Improving global outcomes 2012 clinical practice guideline. Ann. Intern. Med..

[23] Levey AS, Stevens LA (2010). Estimating GFR using the CKD epidemiology collaboration (CKD-EPI) creatinine equation: More accurate GFR estimates, lower CKD prevalence estimates, and better risk predictions. Dam. J. Kidney is. Off. J. Natl. Kidney Found..

[24] Simpson BH, Reith DM, Medlicott NJ, Smith AJ (2018). Choice of renal function estimator influences adverse outcomes with dabigatran etexilate in patients with atrial fibrillation. TH Open Companion J. Thromb. Haemost..

[25] Chan YH (2020). Impacts of different renal function estimation formulas on dosing of DOACs and clinical outcomes. J. Am. Coll. Cardiol..

[26] Nabiee M, Dashti-Khavidaki S, Khajeh B (2020). Dose discordance of direct acting oral anticoagulants using different equations for estimating GFR: A literature review. Exp. Rev. Clin. Pharmacol..

[27] Chan YH (2020). Off-label dosing of non-vitamin K antagonist oral anticoagulants and clinical outcomes in Asian patients with atrial fibrillation. Heart Rhythm.

[28] Raman M, Middleton RJ, Kalra PA, Green D (2017). Estimating renal function in old people: An in-depth review. Int. Urol. Nephrol..

[29] Chin PK (2014). Correlation between trough plasma dabigatran concentrations and estimates of glomerular filtration rate based on creatinine and cystatin C. Drugs R&D.

[30] Lin SY (2020). Real-world rivaroxaban and Apixaban levels in Asian patients with atrial fibrillation. Clin. Pharmacol. Ther..

[31] Steffel J (2018). The 2018 European heart rhythm association practical guide on the use of non-vitamin K antagonist oral anticoagulants in patients with atrial fibrillation: Executive summary. Europace Eur. Pacing Arrhythm. Card. Electrophysiol. J. Work. Groups Card. Pacing rrhythm. Card. Electrophysiol. Eur. Soc. Cardiol..

[32] Lip GY, Nieuwlaat R, Pisters R, Lane DA, Crijns HJ (2010). Refining clinical risk stratification for predicting stroke and thromboembolism in atrial fibrillation using a novel risk factor-based approach: The euro heart survey on atrial fibrillation. Chest.

[33] Pisters R (2010). A novel user-friendly score (HAS-BLED) to assess 1-year risk of major bleeding in patients with atrial fibrillation: The Euro heart survey. Chest.

