# Supplementary material for: Impact of different renal function equations on direct oral anticoagulant concentrations
Source: Sci Rep. 2021 Dec 13;11:23833. doi: 10.1038/s41598-021-03318-4 (PMC8668925; doi:10.1038/s41598-021-03318-4)
Supplement: Supplementary file 1 — Supplementary Information 1. [file 41598_2021_3318_MOESM1_ESM.docx]

*Ultra-high-performance liquid chromatography with tandem mass spectrometry conditions for direct oral anticoagulant concentrations measurement.*

Blood samples were collected through venous puncturing and stored in tubes containing K2EDTA (BD Vacutainer®). The trough concentration was measured right before the next dose of dabigatran, rivaroxaban or appixaban. Blood samples were centrifuged with a standard procedure to obtain plasma and stored in −80°C until use. Plasma dabigatran, rivaroxaban, or apixaban concentration was measured by using ultra-high performance liquid chromatography with tandem mass spectrometry (UHPLC-MS/MS). In total, 100 µL of each plasma sample was deproteinized by adding 400 μL of 100% MeOH and homogenized at 1000 rpm for 2 min in a Geno/Grinder 2010 (SPEX SamplePrep; Metuchen, NJ, USA), followed by centrifugation at 15,000 rpm for 5 min. Thereafter, 400 µL of the supernatant was transferred to a new Eppendorf tube, and the residue was extracted again using the same protocol. The plasma extracts were pooled and dried with a centrifugal vaporizer (Thermo SpeedVac^®^ Savant SPD111V; Thermo Fisher Scientific, Waltham, MA, USA), followed by reconstitution of the sample in 200 μL of MeOH. The reconstituted sample was then filtered through a 0.22-μm polypropylene membrane filter (RC-4; Sartorius, Göttingen, Germany) for injection into a system for ultra-high performance liquid chromatography with tandem mass spectrometry (UHPLC-MS/MS). The instrument used was an Agilent 1290 UHPLC system coupled with an Agilent 6460 triple quadrupole mass spectrometer (Agilent Technologies, Santa Clara, CA, USA). A Kinetex reversed-phase core-shell C18 column (2.1 × 50 mm, 2.6 µm, 100 Å; Phenomenex, Torrance, CA, USA) was used for separation. The mobile phase consisted of 0.1% formic acid in water (solvent A) and 0.1% formic acid in ACN (solvent B). The flow rate was 0.35 mL min^−1^. The gradient profile started with 1% of solvent B for 0.5 min, which was then changed to 15% of solvent B for 0.1 min; this was maintained at 15% of solvent B for 0.9 min, then increased to 100% of solvent B for 0.6 min, which was then maintained for 1.4 min. Finally, the column was re-equilibrated to 1% of solvent B for 1.5 min until the next injection. The temperature of the sample reservoir was maintained at 4°C, and the column oven was set at 40°C. The injection volume was 3 µL. A JetStream electrospray ionizer was employed as the ion source. The MS parameters were set as follows: a 350°C drying gas temperature, 10 L/min drying gas flow rate, 45-psi nebulizer pressure, 350°C sheath gas temperature, an 11-L/min sheath gas flow rate, a 3500-V capillary voltage, and a 500-V nozzle voltage. Mass spectrometry (MS) data acquisition was executed in multiple reaction monitoring mode, and the mass transitions were 472.2→289, 472.2→144 for dabigatran, 478.2→295.1, 478.2→144 for [^13^C_6_]-dabigatran, 436.1→144.9, 436.1→72.9 for rivaroxaban, 442.1→144.9, 442.1→72.9 for [^13^C_6_]-rivaroxaban, 460.2→443.1, 460.2→199 for apixaban, 464.2→447.1, 464.2→203.1 for [^13^C, *d*_3_]-apixaban.

The DOAC concentrations were compared with those in the data reported in clinical trials. The expected trough dabigatran concentration ranged from 31 to 225 ng/mL, the expected trough rivaroxaban concentration ranged from 12 to 137 ng/mL, and the expected range for trough apixaban concentration ranged from 34 to 2301,8,11,12. All plasma drug concentrations were classified to be higher, within, or lower than the expected range, according to the aforementioned value.
