# Supplementary material for: Impact of different renal function equations on direct oral anticoagulant concentrations
Source: Sci Rep. 2021 Dec 13;11:23833. doi: 10.1038/s41598-021-03318-4 (PMC8668925; doi:10.1038/s41598-021-03318-4)
Supplement: Supplementary file 2 — Supplementary Table S1. [file 41598_2021_3318_MOESM2_ESM.docx]

Table S1. Renal function equations to estimate glomerular filtration rate.

| Renal function equation | Abbreviation | Unit | Description | Reference |
| --- | --- | --- | --- | --- |
| Cockcroft–Gault formulae | CrCL | mL/min | $\frac{(140-Age)\times BW\times0.85 (if female)}{72\times CRE}$ | 14 |
| CKD-EPI equation^1^ | CKD-EPI | mL/min | ${135\times min(\frac{CRE}{\kappa}, 1)}^{\alpha}\times{max(\frac{CRE}{\kappa}, 1)}^{-0.601}\times{{min(\frac{CysC}{0.8}, 1)}^{-0.375}\times{max(\frac{CysC}{0.8}, 1)}^{-0.711}\times0.995}^{age}\times0.969 (if female) \times\frac{BSA}{1.73}$ | 20 |
| MDRD Study equation | MDRD | mL/min | $175\times{CRE}^{-1.154}\times{age}^{-0.203}\times0.742 (if female)\times1.212(if black)\times\frac{BSA}{1.73}$ | 22 |
| ^1^*k* is 0.7 for females and 0.9 for males, α is -0.248 for females and -0.207 for males Abbreviations: CKD-EPI equation, Chronic Kidney Disease Epidemiology Collaboration (CKD-EPI) equation; MDRD Study equation, Modification of Diet in Renal Disease Study equation. | | | | |
