# Supplementary material for: Impact of different renal function equations on direct oral anticoagulant concentrations
Source: Sci Rep. 2021 Dec 13;11:23833. doi: 10.1038/s41598-021-03318-4 (PMC8668925; doi:10.1038/s41598-021-03318-4)
Supplement: Supplementary file 3 — Supplementary Table S2. [file 41598_2021_3318_MOESM3_ESM.docx]

Table S2. Basic characteristics for patients with drug concentrations higher than the expected range reported in a clinical study.

| Characteristics | **Dabigatran (n=146)** | | | **Rivaroxaban (n=164)** | | | **Apixaban (n=201)** | | |
| --- | --- | --- | --- | --- | --- | --- | --- | --- | --- |
|  | Higher than range  n=51 | Within or lower than range  n=93 | p-value | Higher than range  n=8 | Within or lower than range  n=156 | p-value | Higher than range  n=11 | Within or lower than range  n=190 | p-value |
| Age (year) | 75.9±8.3 | 71.7±8.8 | 0.007 | 76.3±6.6 | 74.9±7.5 | 0.487 | 81.6±13.5 | 77.0±9.4 | 0.028 |
| Male | 26 (51.0) | 69 (74.2) | 0.005 | 3 (37.5) | 81 (51.9) | 0.488 | 10 (90.9) | 103 (54.2) | 0.025 |
| BW (kg) | 64.1±9.8 | 69.6±11.8 | 0.005 | 68.8±21.4 | 63.1±12.3 | 0.609 | 62.9±9.6 | 63.7±11.5 | 0.873 |
| BMI (kg/m^2^) | 24.5±3.4 | 25.7±3.9 | 0.067 | 26.7±5.6 | 24.6±4.1 | 0.376 | 23.9±3.3 | 24.8±3.7 | 0.413 |
| CRE (mg/dL) | 1.04±0.25 | 0.95±0.21 | 0.019 | 1.31±0.36 | 1.04±0.32 | 0.011 | 1.43±0.37 | 1.15±0.47 | 0.014 |
| Cystatin C (mg/dL) | 1.14±0.34 | 0.98±0.17 | <0.001 | 1.32±0.39 | 1.07±0.34 | 0.022 | 1.34±0.30 | 1.17±0.41 | 0.052 |
| ALT (U/L) | 25.0±21.7 | 20.6±11.4 | 0.164 | 26.0±15.2 | 19.7±11.6 | 0.261 | 29.0±26.2 | 20.7±12.3 | 0.722 |
| eGFR (mL/min) |  |  |  |  |  |  |  |  |  |
| CrCL | 53.2±18.0 | 66.7±18.9 | <0.001 | 40.0±12.1 | 52.5±17.0 | 0.028 | 38.1±17.7 | 49.6±20.1 | 0.046 |
| CrCL<50 | 25 (49.0) | 19 (20.4) | <0.001 | 6 (75.0) | 76 (48.7) | 0.277 | 9 (81.8) | 106 (55.8) | 0.120 |
| CKD-EPI | 63.2±20.0 | 77.3±19.1 | <0.001 | 47.9±13.9 | 65.6±19.3 | 0.001 | 49.3±18.9 | 60.8±21.9 | 0.067 |
| CKD-EPI<50 | 15 (30.0) | 7 (7.6) | <0.001 | 5 (62.5) | 32 (20.5) | 0.015 | 7 (63.6) | 60 (31.7) | 0.045 |
| MDRD | 62.2±19.0 | 77.2±19.5 | <0.001 | 46.0±14.9 | 62.4±18.9 | 0.014 | 50.2±20.9 | 60.6±22.8 | 0.135 |
| MDRD<50 | 13 (25.5) | 6 (6.5) | 0.001 | 5 (62.5) | 45 (28.8) | 0.057 | 6 (54.5) | 62 (32.6) | 0.135 |
| CHA_2_DS_2_VASc^a^ | 4.0±1.4 | 4.0±1.3 | 0.905 | 4.1±1.0 | 3.9±1.6 | 0.542 | 4.9±2.2 | 4.2±1.5 | 0.429 |
| HAS-BLED^b^ | 2.2±0.7 | 2.5±0.8 | 0.025 | 1.9±0.4 | 2.2±0.8 | 0.333 | 2.6±1.1 | 2.4±0.9 | 0.394 |
| Co-morbidities |  |  |  |  |  |  |  |  |  |
| IS or TIA | 19 (37.3) | 69 (74.2) | <0.001 | 0 (0) | 46 (29.5) | 0.107 | 7 (63.6) | 89 (46.8) | 0.358 |
| CHF | 8 (15.7) | 6 (6.5) | 0.074 | 3 (37.5) | 37 (23.7) | 0.405 | 4 (36.4) | 32 (16.8) | 0.112 |
| Hypertension | 39 (76.5) | 72 (77.4) | 0.897 | 7 (87.5) | 117 (75.0) | 0.681 | 7 (63.6) | 150 (78.9) | 0.261 |
| Diabetes | 15 (29.4) | 26 (28.0) | 0.853 | 5 (62.5) | 47 (30.1) | 0.111 | 5 (45.5) | 53 (27.9) | 0.211 |
| MI or PAOD | 2 (3.9) | 6 (6.5) | 0.712 | 0 (0) | 21 (13.5) | 0.598 | 4 (36.4) | 22 (11.6) | 0.039 |
| Malignancy | 8 (15.7) | 9 (9.7) | 0.285 | 0 (0) | 21 (13.5) | 0.598 | 5 (45.5) | 31 (16.3) | 0.014 |
| Bleeding history | 4 (7.8) | 15 (16.1) | 0.203 | 0 (0) | 19 (12.2) | 0.598 | 4 (36.4) | 33 (17.4) | 0.122 |
| ICH | 0 (0) | 4 (4.3) | 0.297 | 0 (0) | 2 (1.3) | 1.000 | 0 (0) | 9 (4.7) | 1.000 |
| GI bleeding | 2 (3.9) | 5 (5.4) | 1.000 | 0 (0) | 8 (5.1) | 1.000 | 3 (27.3) | 11 (5.8) | 0.032 |
| Other bleeding | 1 (2.0) | 7 (7.5) | 0.260 | 0 (0) | 11 (7.1) | 1.000 | 1 (9.1) | 13 (6.8) | 0.558 |
| Trough concentration | 365.8±158.2 | 90.9±57.0 | <0.001 | 245.9±72.7 | 33.8±30.9 | <0.001 | 226.9±49.1 | 87.1±43.0 | <0.001 |
| Medication use^c^ |  |  |  |  |  |  |  |  |  |
| Standard dose | 13 (27.7) | 18 (18.6) | 0.213 | 5 (62.5) | 84 (53.8) | 0.728 | 3 (27.3) | 83 (43.7) | 0.358 |
| Reduced dose | 34 (72.3) | 79 (81.4) |  | 3 (37.5) | 72 (46.2) |  | 8 (72.7) | 107 (56.3) |  |
| Poor adherence^d^ | 2 (3.9) | 12 (14.0) | 0.081 | 1 (12.5) | 8 (5.3) | 0.381 | 0 (0) | 28 (15.6) | 0.363 |
| Concurrent medications^e^ |  |  |  |  |  |  |  |  |  |
| amiodarone | 11 (21.6) | 15 (16.1) | 0.417 | 3 (37.5) | 25 (16.0) | 0.138 | 6 (54.5) | 51 (26.8) | 0.047 |
| dronedarone | 1 (2.0) | 0 (0) | 0.354 | 2 (25.0) | 5 (3.2) | 0.039 | 1 (9.1) | 9 (4.2) | 0.404 |
| verapamil | 0 (0) | 2 (2.2) | 0.539 | 0 (0) | 0 (0) | -- | 1 (9.1) | 6 (3.2) | 0.330 |
| NSAID | 3 (5.9) | 3 (3.2) | 0.666 | 0 (0) | 5 (3.2) | 1.000 | 0 (0) | 1 (0.5) | 1.000 |
| aspirin | 0 (0) | 4 (4.3) | 0.297 | 0 (0) | 2 (1.3) | 1.000 | 0 (0) | 4 (2.1) | 1.000 |
| clopidogrel | 1 (2.0) | 2 (2.2) | 1.000 | 0 (0) | 3 (1.9) | 1.000 | 1 (9.1) | 4 (2.1) | 0.247 |
| Data are expressed as mean ± standard deviation or number (percentage). A total of 146 participants were enrolled to dabigatran group and contributed 144 dabigatran trough concentrations, and all participants in rivaroxaban and apixaban groups contributed trough concentrations.  ^a^CHA2DS2VASc score: To evaluate the risk for ischemic stroke among patients with atrial fibrillation. Higher score indicates higher risk of ischemic stroke. For CHA2DS2VASc score, the additional risk factors including assigning one point to age 65-74 years, female sex, or vascular disease and two points to age ≥75 years.  ^b^HASBLED score: To evaluate the risk for bleeding. Higher score indicates higher risk. One point is assigned to hypertension, abnormal liver function, abnormal renal function, stroke history, bleeding history, labile international normalized ratio (INR) during warfarin therapy, age over 65 years, antiplatelet agent, non-steroidal anti-inflammatory drug or ethanol use. The item labile INR was not calculated in the present study.  ^c^Standard dose: 150 mg twice daily for dabigatran, 15 mg daily for rivaroxaban, and 5 mg twice daily for apixaban; reduced dose: 110 mg twice daily for dabigatran, 10 mg daily for rivaroxaban, and 2.5 mg twice daily for apixaban.  ^d^Poor adherence was defined as no self-reported missed dabigatran dose during 7 days before drug concentration monitoring. A total of 8 patients had missed data.  ^e^Concurrent medications: None of the participants used azole antifungal agents, protease inhibitors (P-glycoprotein inhibitors), and rifampin, enzyme inducing antiepileptic drugs such as phenytoin and phenobarbital (P-glycoprotein inducers).  **Abbreviations:** BMI, body mass index; BW, body weight; CKD-EPI, glomerular filtration rate estimated by using the Chronic Kidney Disease Epidemiology Collaboration Equation featured both creatinine and cystatin C; CHF, congestive heart failure; CrCL, creatinine clearance estimated by using the Cockroft–Gault formula; CRE, serum creatinine; DE, dabigatran etexilate; eGFR, estimated glomerular filtration rate; ICH, intracranial hemorrhage; IS, ischemic stroke; MDRD, the GFR estimated by using the Modification of Diet in Renal Disease (MDRD) Study equation; MI, myocardial infarction; NSAID, non-steroidal anti-inflammatory drugs; PAOD, peripheral arterial vascular disease; TIA, transient ischemic attack. | | | | | | | | | |
